# Immunogenicity of a novel anti-allergic vaccine based on house dust mite purified allergens and a combination adjuvant in a murine prophylactic model

**DOI:** 10.3389/falgy.2022.1040076

**Published:** 2022-11-17

**Authors:** Wendy Ramírez, Damarys Torralba, Virgilio Bourg, Miriam Lastre, Oliver Perez, Alain Jacquet, Alexis Labrada

**Affiliations:** ^1^ Department of Allergens, Allergens Lab, Centro Nacional de Biopreparados (BIOCEN), Bejucal, Mayabeque, Cuba; ^2^ Department of Immunology, Havana University of Medical Sciences, Havana, Cuba; ^3^ Department of Biochemistry, Faculty of Medicine, Chulalongkorn University, Bangkok, Thailand

**Keywords:** allergy immunotherapy, *Dermatophagoides siboney*, combination adjuvant, TLR ligands, adjuvanted vaccine

## Abstract

The outer-membrane-derived proteoliposome (PL) of *Neisseria meningitidis* has been reported as a potent vaccine adjuvant, inducing a Th1-skewed response. This work aimed to assess the immunogenicity of a novel anti-allergic vaccine candidate based on allergens from *Dermatophagoides siboney* house dust mite and a combination adjuvant containing PL and Alum. In a preventative experimental setting, BALB/c mice were administered with three doses containing 2 µg of Der s1 and 0.4 µg Der s2 allergen, PL and Alum, at 7 days intervals, by subcutaneous route. Furthermore, mice were subjected to an allergen aerosol challenge for 6 consecutive days. Serum IgE, IgG1, and IgG2a allergen-specific antibodies were assessed by ELISA. Cytokine levels in supernatants of *D. siboney* stimulated lymphocyte cultures and in bronchoalveolar lavage (BAL) were measured by ELISA. Lung tissues were subjected to histological examination. The vaccine prevented the development of both, systemic (IgE) and local allergic responses (featuring lower IL-4, and IL-5 levels in BAL) upon allergen exposure by the inhalant route. Histological examination showed also a diminished allergic inflammatory response in the lungs. After the allergen challenge, cytokine levels in stimulated lymphocyte cultures showed lower values of IL-13 and augmented IFN-γ and IL-10. The vaccine induced a mixed IgG2a/IgG1 antibody response; although only IgG2a was PL-dependent. Both, IgG1/IgE and IgG2a/IgE ratios, showed significantly greater values in vaccinated mice. The findings support a preventative anti-allergic effect associated with the induction of a Th1-like IFN-γ/IL-10 response. IgG1/IgE and IgG2a/IgE ratios could be useful biomarkers for translation into clinical trials.

## Introduction

Allergen-specific immunotherapy (AIT) is based on repeated administration of allergen injections intended to achieve immune tolerance towards the offending allergen. Hallmarks of the allergic response are an exaggerated allergen-specific IgE antibody response and high local production of Th2 inflammatory cytokines, particularly IL-4, IL-5, and IL-13. The development of more effective AIT vaccines aimed to overturn the exacerbated Th2 immunity is a present-day problem. Strategies based on promoting Th1 or Treg responses are beneficial in allergic humans and animal models of allergic diseases (1–3). Adjuvants for allergen vaccines based on agonists for pattern recognition receptors, including Toll-like receptors (TLRs), are promising (4–6). Their immunostimulatory activity can be improved if they are combined with appropriate delivery systems (4–7).

Alum adjuvants have been in use for human vaccines for almost 100 years. Alum increases antigen uptake, reduces antigen degradation, and sustains antigen presentation by dendritic cells. On the other hand, Alum is known to potently induce IgG4 blocking antibodies (8, 9) with a beneficial effect against allergic disease. Moreover, recent evidence indicates that Alum can influence the innate immune environment, activating the NALP inflammasome and the release of proinflammatory cytokines IL-1β and IL-18 (10, 11). However, its potential pro-Th2 adjuvant activity is a caveat for Alum-adjuvanted allergen vaccines. The combination of Alum with TLR agonists might dampen Alum's pro-Th2 activity and improve the efficacy of allergen vaccine formulations (3, 7, 10, 12).

The outer membrane vesicle or proteoliposome (PL) from *Neisseria meningitidis* serogroup B has been reported as a nontoxic and potent vaccine adjuvant, for an anti-meningococcal vaccine. PL induces a Th1-skewed response (13, 14) and boosts the maturation of dendritic cells (15). The immunomodulatory activity of the *N. meningitidis* PL is known to reside mostly on its TLR4-binding activity, linked to its LPS content, a well-known TLR4 ligand (13, 15). TLRs as vaccine adjuvants can have anti-allergic action providing a combination of enhanced Th1 deviation, regulatory responses, and induction of blocking antibodies (16).

The use of PL as adjuvant in allergen-specific vaccines could be a valuable alternative to the current AIT based on aqueous or alum-absorbed allergen extracts (7, 17, 18). In previous studies, a novel anti-allergic vaccine formulation based on purified allergens from *Dermatophagoides siboney* house dust mite (HDM) and PL-Alum adjuvant combination was tested in animal models showing no signs of direct toxicity or functional immunoallergic toxicity, at dose levels greatly in excess to those proposed for early phase clinical trials (19). A major potential benefit provided by this adjuvanted vaccine would be a decrease in the number of injections required for conventional AIT and the treatment length, as well as, the amount of injected allergen, improving its safety (13).

The present study aimed to assess the immunogenicity of a novel anti-allergic vaccine candidate based on purified allergens from *D. siboney* HDM together with a combination adjuvant containing *N. meningitidis* PL and Alum.

## Methods

### Substances and reagents

*Allergen:* A purified fraction of the HDM *D. siboney* allergen extract was used for administration to mice. This fraction, named DS1/DS2, contains both major allergens, Der s 1 and Der s 2 at a proportion of 5:1. It was obtained by a purification process in Good Manufacturing Practices (GMPs) conditions (20), starting from the standardized freeze-dried allergen extract VALERGEN-DS (BIOCEN, Cuba) (21).

*Vaccine composition*: The preparation contained the DS1/DS2 purified fraction of *D. siboney* (8 µg/ml Der s 1 and 1.6 µg of Der s 2), 100 µg/ml of *N. meningitidis* serogroup B PL (Finlay Vaccine Institute, Havana) (13, 22), and 2 mg/ml Aluminium Hydroxide gel (CRODA, Denmark). The preparation was formulated in phosphate-buffered saline (PBS, pH 7.2); Thiomersal (0.1% w/v) has been added as a preservative. The vaccine batch was prepared in GMP conditions at BIOCEN (Bejucal, Cuba).

All other chemicals/reagents used throughout these studies were obtained from Sigma (St. Louis, MO) unless otherwise noted.

### Animals

This study was carried out following the national regulation No. 64/13, of the Cuban Centre for the State Control of Medicines, Equipment and Medical Devices (CECMED, Spanish acronyms). The protocol was approved by the Institutional Committee for Care and Use of Laboratory Animals of BIOCEN (23).

Clinically healthy specific pathogen-free BALB/c mice, aged 6–8 weeks, were purchased from CENPALAB (Havana, Cuba). Animals were housed under controlled environmental conditions (i.e., 21 ± 3 °C; 40%–70% relative humidity, 12 h light/dark cycle) and *ad libitum* access to water and food.

#### Experimental design for assessing the preventative anti-allergic effect

BALB/c mice were distributed into four groups, 10 mice/group, 5 males and 5 nulliparous, and non-pregnant females in each group. The mice were given three doses of the vaccine (at 7-day intervals) of 0.25 ml each, injected by sc route in the dorsal region. The “Th2 control” group was injected with a formulation of the allergen adsorbed onto aluminium hydroxide (8 µg Der s 1/ml plus 1.6 µg/ml Der s 2 in 2 mg/ml Alum) intended to induce a typical Th2 allergic response. Two other non-allergen placebo control groups were injected with PBS or Alum (2 mg/ml). One week after the last dose, the mice were subjected to the aerosol allergen challenge. The same consisted of repeated exposure (30 min/day during 7 consecutive days) to the aerosolized allergen in a whole-body chamber. For this, an MPC Aerosol Medication Nebulizer (Braintree Scientific, Braintree, MA) was connected to a plastic chamber where the mice were kept during the challenge (24). The allergen solution used for nebulization was the same allergen mix DS1/DS2 (500 µg/ml Der s 1 (25), 100 µg Der s 2 (as measured by specific Der f 2 ELISA kit; Indoor Biotechnologies, Charlottesville, VA)). After taking into account, both starting concentration and chamber volume, the actual allergen concentration in air was calculated as 105 µg/L Der s 1 and 21 µg/L Der s 2.

At 48 h after the final aerosol (i.e., day 30), mice were euthanized by cervical dislocation. Then, lymph node cells were isolated to evaluate the specific T cell response in lymphoproliferative assays.

Blood samples were collected from the retro-orbital plexus for serum antibody determinations at days 0, 14 (1 week after two doses), 21 (1 week after three doses), and 28 (after the final aerosol challenge).

#### Experimental design for assessing the effect of pre-existing immunity to PL

Another experiment in mice was performed aiming to assess the influence of a previously established immune response to *N. meningitidis* on the allergen-specific response. For this purpose, BALB/c mice were distributed in three groups, 10 mice/group (5 males and 5 nulliparous and non-pregnant females in each group). The mice were primed by giving two doses of PL in Alum (100 µg/ml PL, 2 mg/ml Alum) at days 0 and 14. Three weeks later, the mice were injected with three doses of the allergen vaccine (allergen plus PL-Alum at 7-day intervals) at days 37, 44, and 51. Two control groups were used; one received only the allergen vaccine, and the other, only the PL priming. Blood samples from the retro-orbital plexus were collected for serum antibody determinations 1 week after the priming (day 22) and 1 week after the last immunization (day 58), then, the mice were euthanized by cervical dislocation.

### Allergen-specific antibody testing

An indirect ELISA was used for the quantification of serum allergen-specific IgE, IgG1, and IgG2a levels. Microtitter plates (Maxisorp Nunc, Copenhagen, Denmark) were coated overnight at 4 °C with 2000 BU/ml of *D. siboney* allergenic extract (VALERGEN, BIOCEN, Cuba) in carbonate buffer pH 9.6. The non-specific binding was blocked with 1% PBS-T (PBS + 0.05% Tween 20)-BSA (bovine serum albumin 10 mg/ml) for 1 h at 37 °C. After incubating 2 h with the samples, the plates were incubated with anti-mouse IgG1 and IgG2a biotin conjugates (1:1000) or anti-mouse IgE Horseradish Peroxidase conjugate (1:1000) for 1 h at 37 °C in blocking solution. A range of dilutions was explored for IgE (1:5 to 1:100) and IgG1 or IgG2a (1:10 to 1:1000) and the dilution lying in the linear region of the dose-response curve was selected for comparing between groups (1:20 for IgE and IgG2a and 1:100 for IgG1). For biotin-antibody conjugates, Streptavidin-Peroxidase 1:2000 was added and incubated for 1 h at 37 °C. Colour was developed by the addition of 100 µl 3,3′,5,5′-tetramethylbenzidine (TMB) solution. Then, the optical density (OD) in each well was measured at 450 nm in a PR521 plate reader (SUMA, Havana). All results were expressed in OD values.

### *N. meningitidis* antibody test

An indirect ELISA was used for the quantification of serum anti-*N. meningitidis* IgG1 and IgG2a levels. Microtiter plates (Maxisorp Nunc, Copenhagen, Denmark) were coated overnight at 4 °C with 100 µ/ml of PL in carbonate buffer [pH 9.6]. The non-specific binding was blocked with 1% PBS-T (PBS + 0.05% Tween 20)-BSA [10 mg/ml]) for 1 h at 37 °C. After incubating 2 h with test sera (1:100), the plates were incubated with anti-mouse IgG1 or IgG2a biotin conjugates and the test continued as described above.

### Blood eosinophils

Eosinophil levels (EOS) in peripheral blood were analysed using eosin stain 1% (Quimefa, Cuba) and counted in a Newbauer chamber; results were expressed as EOS/ml blood.

### Lymphocyte response

Cells from draining lymph nodes from the head–neck region (cervical, axillary, and brachial) were collected and cultured to evaluate the specific cellular response regarding cytokine production (IL-13, IL-10, and IFN-γ) as stimulated with the allergen. Mouse lymph nodes were aseptically removed 48 h after the final aerosol challenge and placed in sterile dishes containing RPMI 1640 media. Single-cell suspensions were prepared by gently pressing the spleen through sterile 40 μm nylon cell strainers (BD Biosciences, San Jose, CA, USA). The cells were cultured (4 × 10^6^ cells/well) in RPMI medium supplemented with HEPES buffer 25 mmol/L, 2 mmol/L L-glutamine, 50 μg/ml gentamicin sulphate, and 0.5% fresh-frozen mouse serum, at 37 °C and 5% CO_2_ atmosphere for 72 and 90 h. The culture was stimulated with DS1/DS2 purified fraction at a concentration of 10 μg/ml Der s 1; 2 µg/ml Der s 2. PL and Phytohaemagglutinin (PHA) (5 µg/ml) were used as positive controls for proliferation checking, whereas the negative control was the non-stimulated culture. IL-13, IL-10, and IFN-γ levels in the antigen-stimulated culture supernatant were measured by a sandwich kit ELISA according to the manufacturer's instructions (Bender MedSystems, USA).

### Cytokine levels in bronchoalveolar lavage fluid (BAL)

The mice were anaesthetized by intraperitoneal injection of Diazepam and Ketamine solution (Labsynth, Brazil). The trachea was cannulated, and the lungs were gently washed twice with a total volume of 1.0 ml ice-cold PBS. BAL was immediately centrifuged at 2000 rpm for 5 min, and the collected supernatant was stored at −70 °C. Levels of cytokines (IL-4, IL-5, IL-10, and IFN-γ) in BAL were measured by a sandwich ELISA kit according to the manufacturer's instructions (Bender MedSystems, USA). Values were calculated using the standard curve provided by the manufacturer and expressed in pg/ml.

### Histological examination of lung tissues

Lung tissues of 5 mice/group were removed and fixed in 10% (v/v) neutral-buffered formalin, then dehydrated, embedded in paraffin, and cut into 4-μm sections that were deparaffinized with xylene, then, stained with hematoxylin and eosin (H & E). Stained sections were analysed under an optical microscope.

### Statistical analysis

Between-group comparisons were performed by 1-way ANOVA supplemented by the Tukey test or 2-way ANOVA supplemented by a Bonferroni test when comparing between groups and dates. All evaluations were carried out using Prism v.5.0 software (GraphPad Inc., San Diego, CA, USA).

## Results

### Allergen-specific antibody response induced by the vaccine

**Figure 1:**
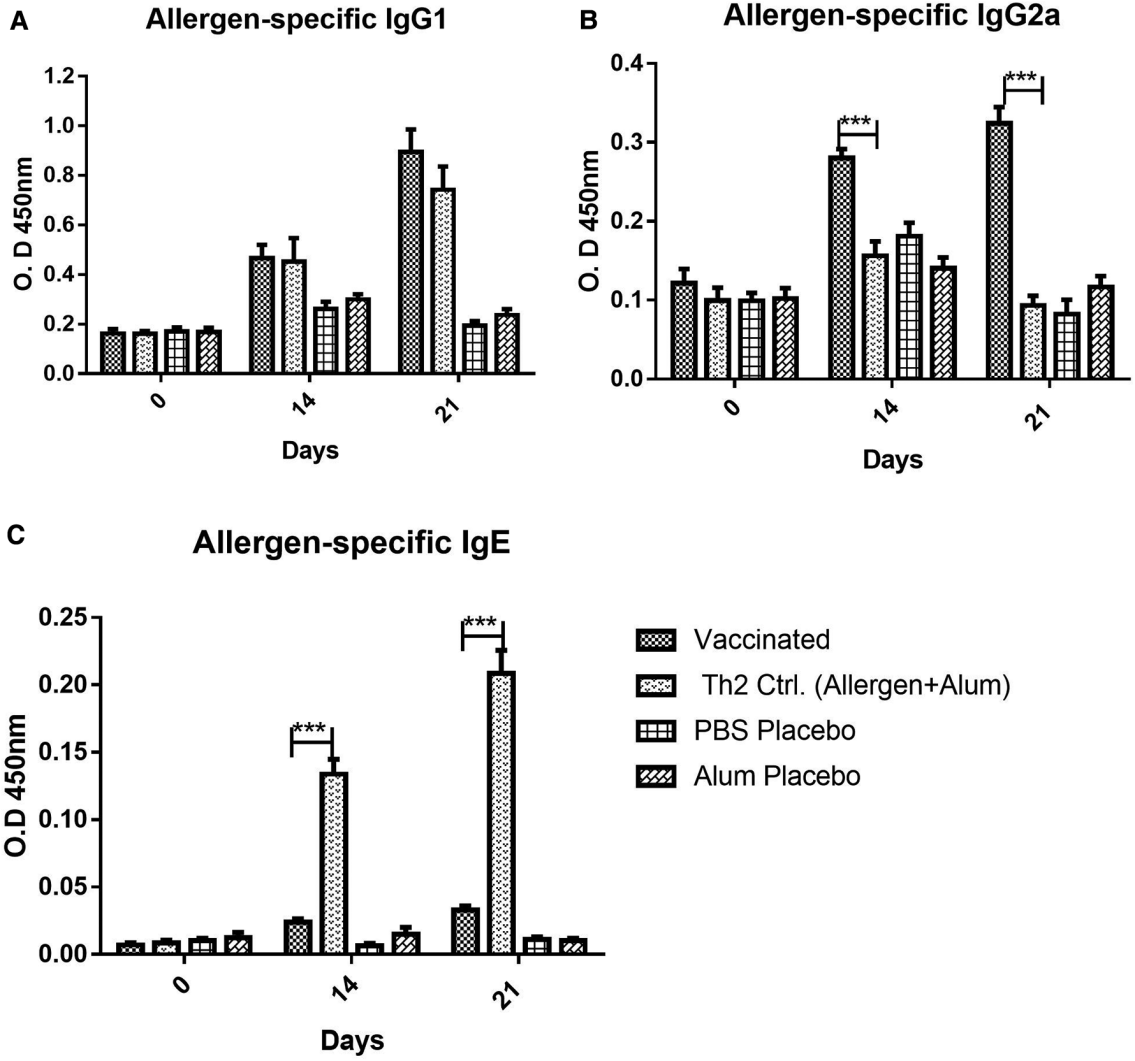
Allergen-specific antibody response, (**A**) IgG1, (**B**) IgG2a, (**C**) IgE, to vaccine and placebos (alum in PBS or PBS alone), as well as, in the Th2 control group injected with an allergen formulation adsorbed into alum lacking the PL adjuvant, at 0, 14, and 21 days after the beginning of the immunization. Values are shown as mean ± SD from *n* = 10/group. (***p < 0.001; 2-way ANOVA, Bonferroni test).

The antibody response upon vaccination, as measured by allergen-specific ELISA, showed a significant increase of IgG allergen-specific antibodies, both, of IgG1 (Figure 1A) and IgG2a (Figure 1B) subclasses, after two or three doses. Regarding IgG1, strong antibody response was noted in vaccinated mice, featuring similar values to the Th2 control group sensitized by allergen injections plus Alum (i.e., lacking PL). After three doses, the IgG1 response peaked and was significantly higher in the vaccine group as compared with placebos (*p* < 0.001, 2-way ANOVA, Bonferroni test), whereas no significant difference was observed as compared with the Th2 control. Therefore, the addition of the PL adjuvant to the vaccine was not a factor influencing the IgG1 allergen-specific response.

In contrast, the IgG2a response (Figure 1B) was remarkably influenced by the inclusion of PL in the vaccine composition. Even at 14 days (after the second dose), the response in the vaccinated groups was significantly greater as compared with both placebos and the Th2 control (*p* < 0.001, 2-way ANOVA, Bonferroni test). This Th2 control group, as expected, absolutely failed to mount an IgG2a response, confirming the PL-dependence of the IgG2a response, as an indicator of a Th1 bias.

In addition to IgG subclasses, the vaccine administration also induced a minor allergen-specific IgE response, although the antibody levels in the vaccinated mice were very low as compared with the Th2 control (*p* < 0.001) (Figure 1C), both after two or three doses.

As noted, in the IgG1, IgG2a, and IgE antibody response, no significant differences were found between placebo groups administered with PBS or Alum. For this reason, in the rest of the experiments, only the results with the PBS placebo are shown to simplify the charts.

### Antibody response after the allergen challenge

**Figure 2:**
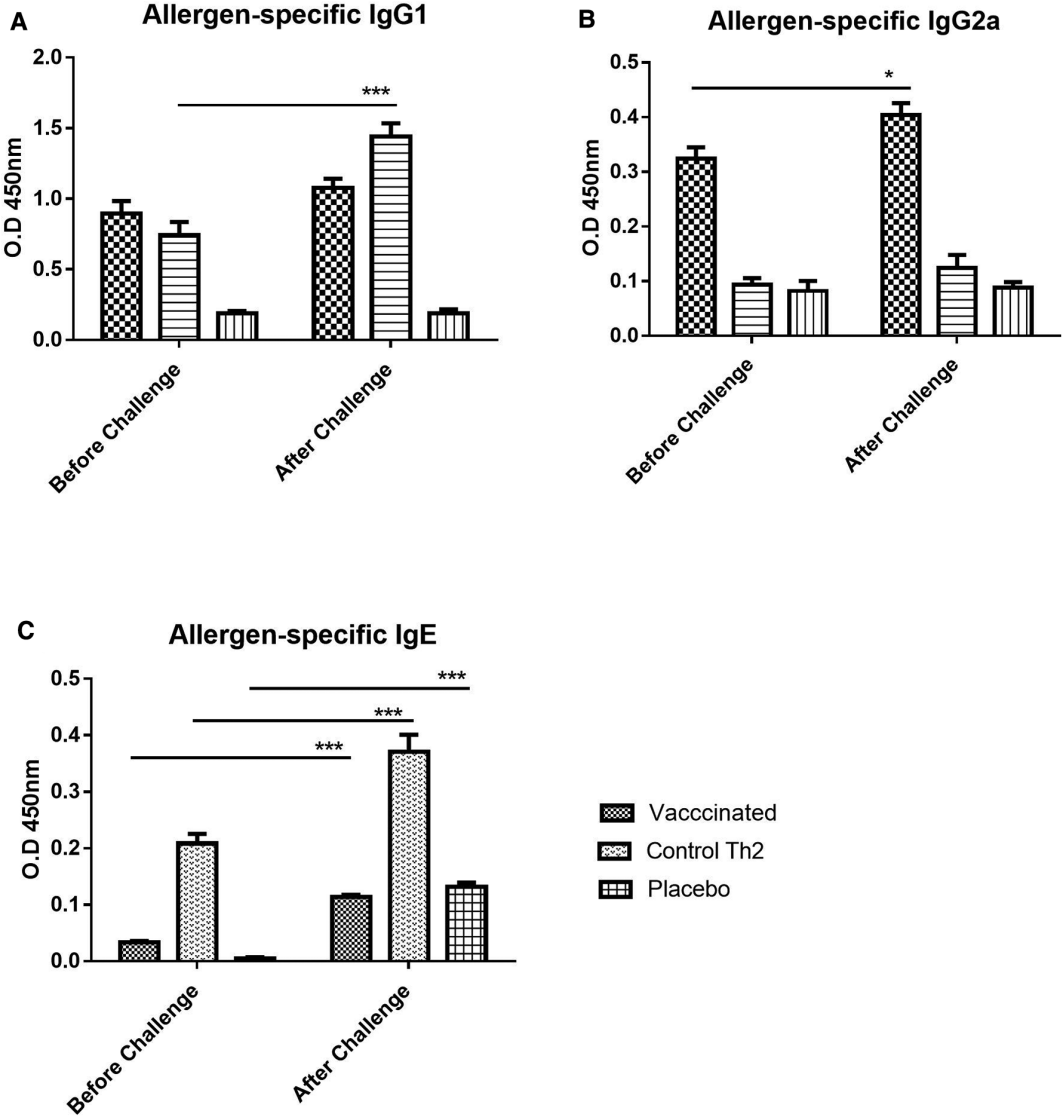
Allergen-specific antibody response upon allergen challenge: before (at 21 days) and after (at 28 days) the challenge. (**A**) IgG1, (**B**) IgG2a, (**C**) IgE. Values are shown as mean ± SD from *n* = 10/group. (*p*-values from 2-way ANOVA, Bonferroni test). The vaccinated mice showed an increase of IgG1 as well as a moderate increase of IgE, the Th2 control showed an exacerbated IgE response. PBS was used as the placebo. (*p < 0.05; ***p < 0.001; 2-way ANOVA, Bonferroni test).

In addition to the primary antibody response upon the vaccine injection in naïve mice, a secondary response after exposing mice to the allergen aerosol challenge was also measured, aiming to assess protection against the occurrence of allergic reactions triggered by allergen exposure. Overall, both IgG1 and IgG2a profiles remained similar after the challenge (Figures 2A,B). IgG1 values apparently tended to increase slightly, both in the vaccine group and in the Th2 control, although no significant change was achieved. As noted before for the primary response, the IgG2a antibodies were raised only in the vaccine group and were kept unchanged after the challenge.

In contrast to IgG, allergen-specific IgE antibodies did show a remarkable exacerbation response to the aerosol challenge. Nevertheless, IgE was significantly diminished in the vaccine group as compared with the Th2 control group (Figure 2C, 2-way ANOVA, Bonferroni post-tests, *p* < 0.001), both, before and after the challenge. Although an increase in allergen-specific IgE upon challenge was noted for both groups, it was much lower for the vaccine group (2-way ANOVA, *p* < 0.001 for Th2 control, *p* < 0.01 for the vaccine group). The placebo group, i.e., mice injected only with placebo and challenged with the allergen showed also, as expected, a significant increase (2-way ANOVA, *p* < 0.001) of IgE, indicating an incipient sensitization process.

**Figure 3:**
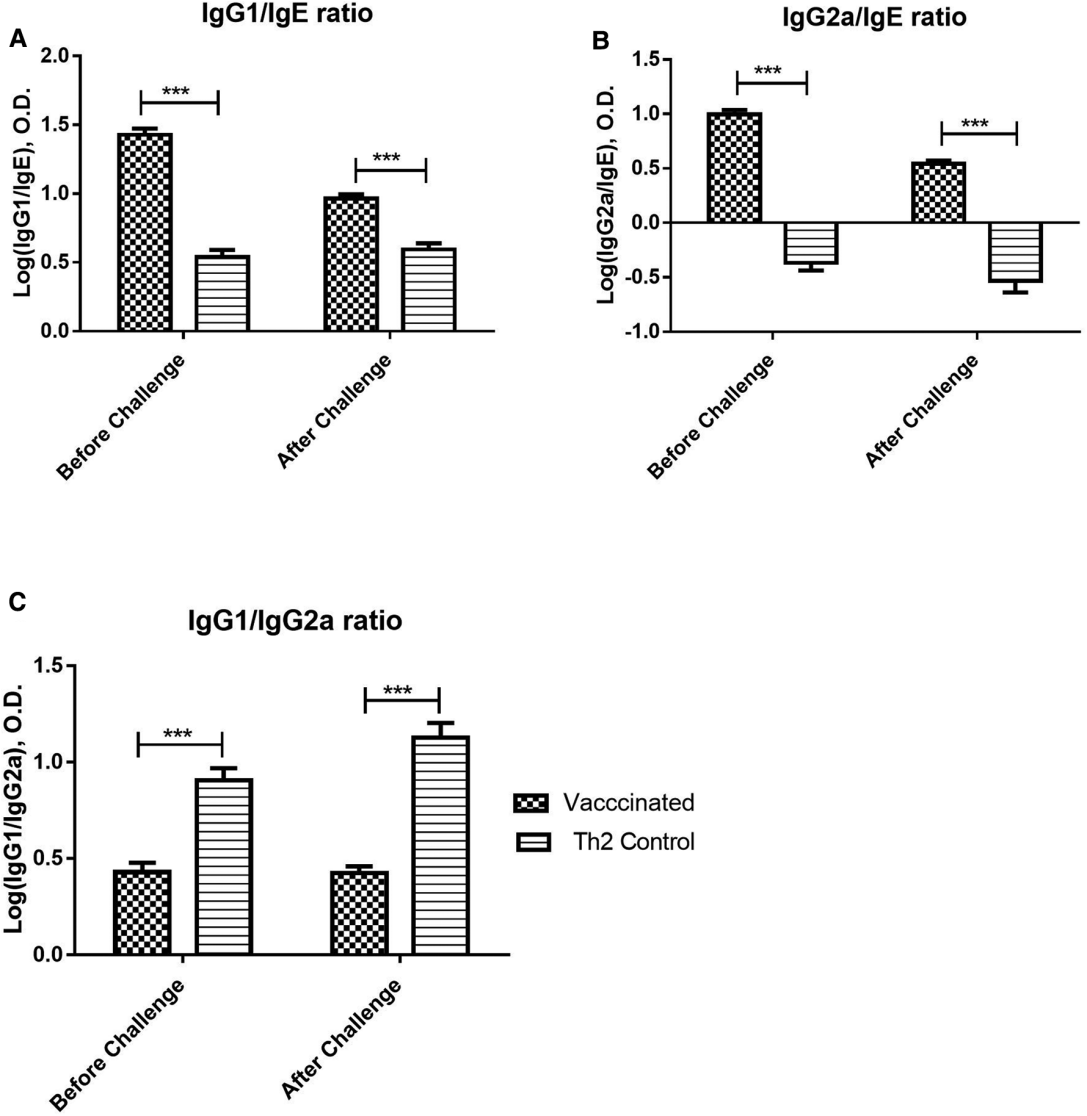
Allergen-specific IgG/IgE ratios and IgG1/IgG2a ratio upon allergen challenge: before (at 21 days) and after (at 28 days) the challenge. Values are shown as mean ± SD from *n* = 10/group. (*p*-values from 2-way ANOVA, Bonferroni test). (**A**) Allergen-specific IgG1/IgE ratio, (**B**) Allergen-specific IgG2a/IgE ratio, (**C**) Allergen-specific IgG1/IgG2a ratio. The vaccinated mice showed a significant difference as compared with the Th2 control group prior to the challenge and kept this significant difference after the challenge. (***p < 0.001; 2-way ANOVA, Bonferroni test).

The IgG/IgE ratio can be indicative of protective immunogenicity in a murine model of respiratory allergy and combines in a single variable both potentially protecting and harmful effects of the immune response. Both, IgG1/IgE and IgG2a/IgE ratios (Figures 3A,B), were significantly greater in vaccinated mice before the allergen challenge, as compared with the Th2 control group, and maintained this favourable difference after the challenge (2-way ANOVA, *p* < 0.001). On the other hand, the IgG1/IgG2a ratio can be indicative of Th2/Th1 balance. The IgG1/IgG2a ratio (Figure 3C) was significantly diminished in vaccinated mice as compared with the Th2 control group, both, before and after the allergen challenge, indicating an advantage of the vaccine over the alum-adjuvanted formulation without PL (2-way ANOVA, *p* < 0.001).

### Eosinophils in blood after the allergen challenge

**Figure 4:**
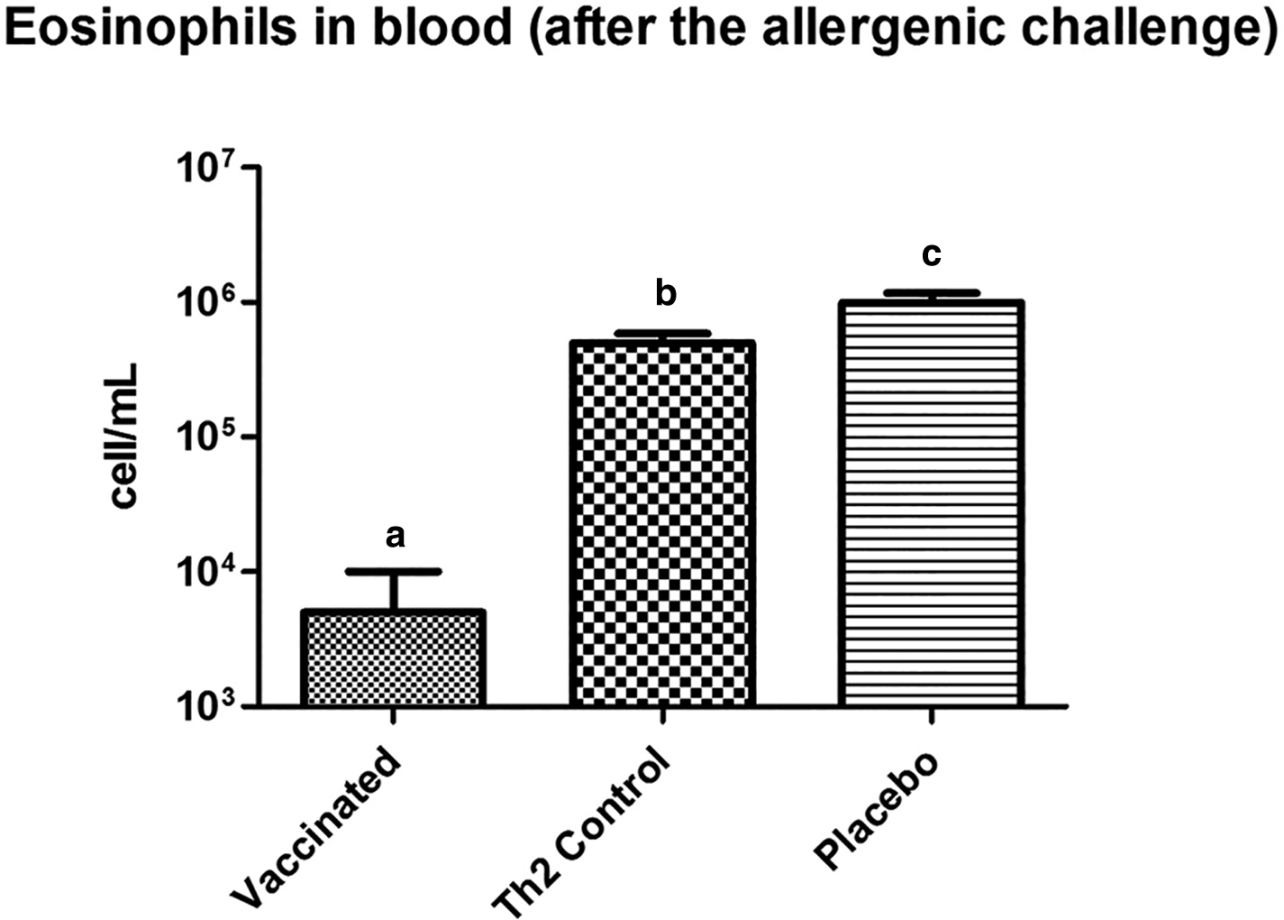
Eosinophil counting in peripheral blood, in mice subjected to the allergenic challenge. Vaccinated mice show a diminished eosinophil response. Letters a, b, and c indicate significant differences. PBS was used as the placebo. Values are shown as mean ± SD from *n* = 10/group (*p* < 0.05, ANOVA, Tukey test).

Prior to the allergen challenge, blood eosinophil levels in vaccinated mice did not differ from those in either of the two control groups (data not shown). However, after the allergen exposure, vaccinated mice showed significantly lower values (125-fold less) of eosinophils as compared with the Th2 control mice (Figure 4).

### Cytokine levels in supernatants of lymphocyte cultures

**Figure 5:**
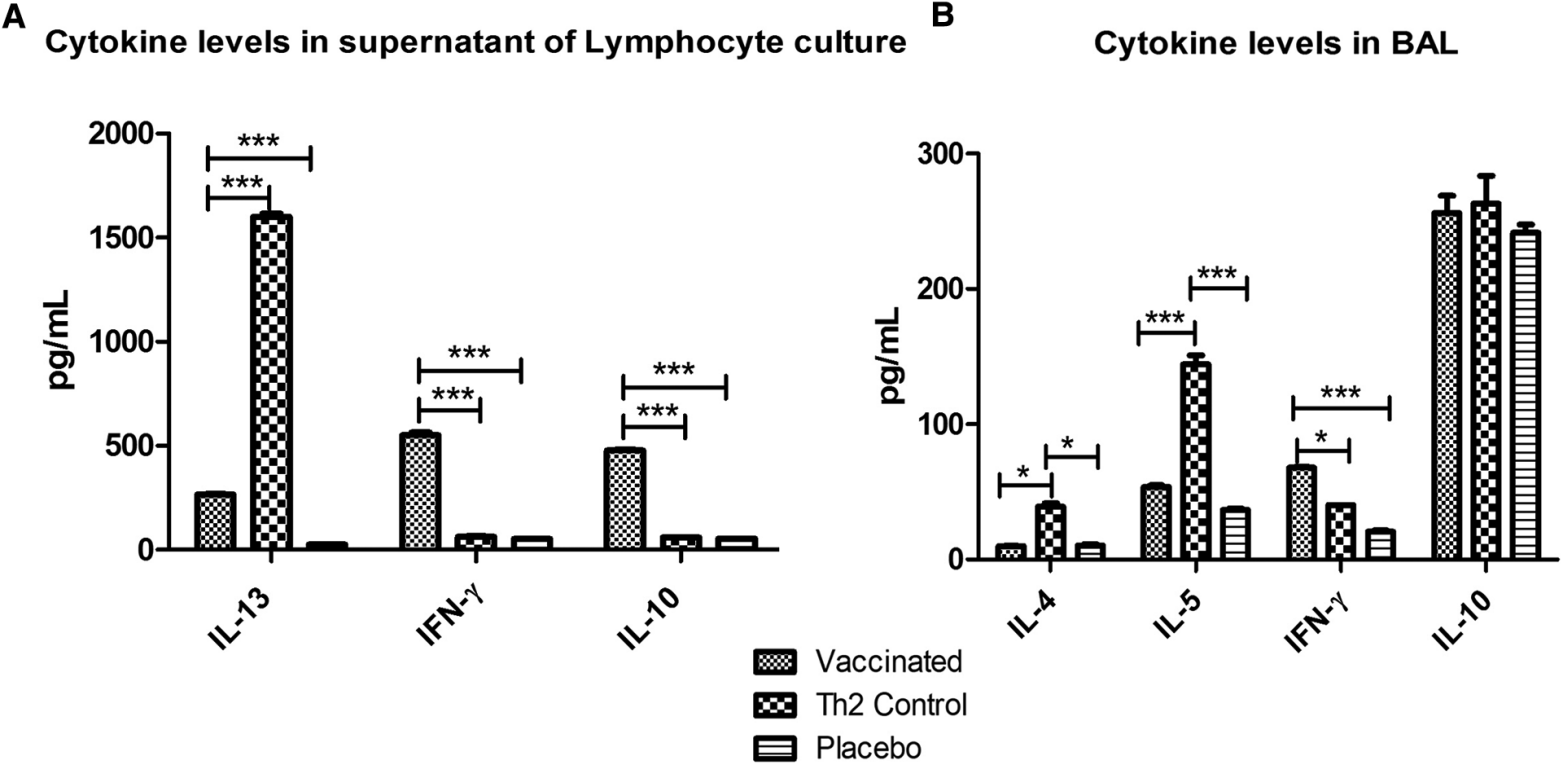
Cytokine levels after allergen challenge in supernatants of lymphocyte cultures stimulated with the allergen (**A**) and in BAL (**B**). In lymphocyte culture, vaccinated mice showed augmented values of IFN-γ and IL-10 and diminished IL-13 levels as compared with the Th2 control group. In BAL, vaccinated mice showed also increased IFN-γ levels and decreased Th2 inflammatory cytokines (IL-4 and IL-5). PBS was used as the placebo. Values are shown as mean ± SD from *n* = 5/group. (*p < 0.05; ***p < 0.001; 2-way ANOVA, Bonferroni test).

Cytokine levels in supernatants of lymphocyte cultures stimulated with the allergen showed significant lower values of IL-13 (*p* < 0.001) in the vaccinated group as compared with the Th2 control group, upon allergen challenge; whereas IFN-γ and IL-10 were significantly (*p* < 0.001) greater. Whereas no significant differences were noted between the Th2 control and PBS placebo groups (Figure 5A) regarding IFN-γ and IL-10.

### Local inflammatory response upon allergen exposure

The cellular inflammatory response in the lungs after the allergen challenge was assessed by measuring marker cytokines in BAL by ELISA (Figure 5B). In the vaccinated group, the Th2 cytokines involved in allergic inflammation were significantly diminished (IL-4, *p* < 0.05 and IL-5, *p* < 0.001) as compared with the Th2 control group, whereas no significant differences were noticed as compared with the placebo group. IFN-γ was significantly augmented only in the vaccinated group (*p* < 0.05 regarding the Th2 control group and *p* < 0.001 regarding the placebo group). No differences were observed among groups in IL-10 levels.

**Figure 6:**
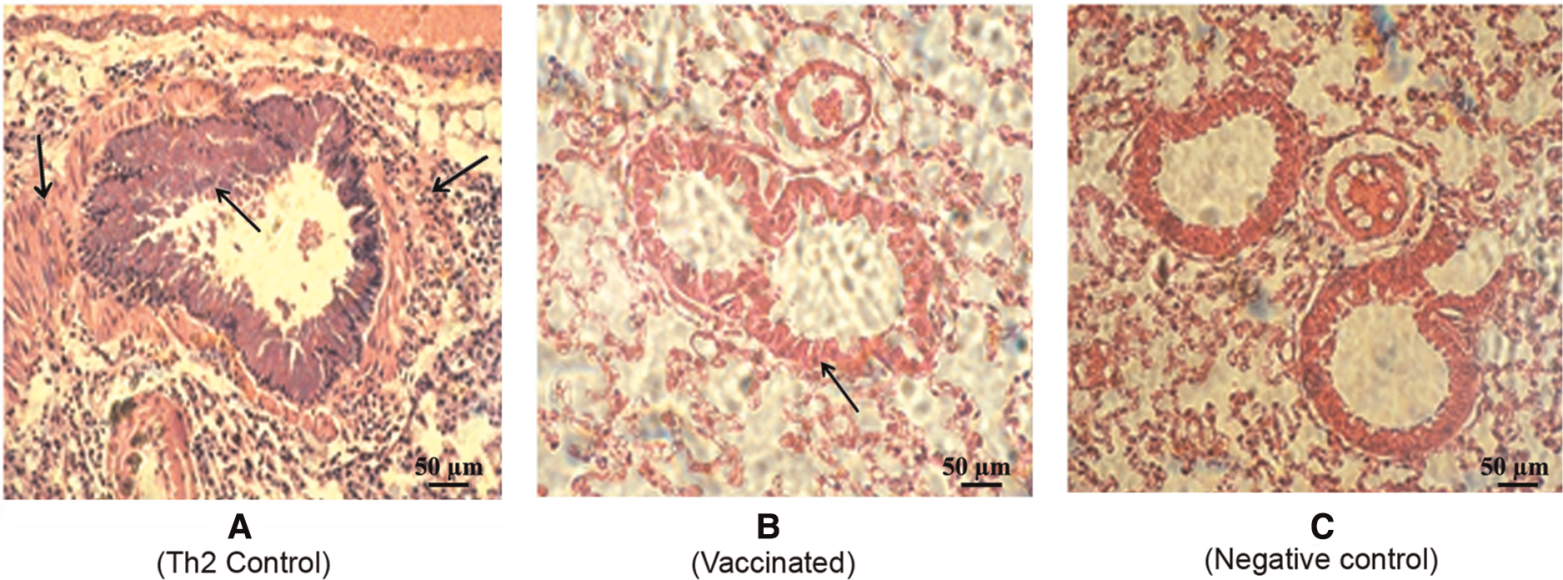
Representative lung histology sections after the allergen challenge. The lungs were stained by hematoxylin-eosin (200×). (**A**) Th2 control group depicting allergic response with peribronchial inflammation characterized by bronchial hyperreactivity features (defined by bronchoconstriction and stenosis with hyperplasia of the epithelial cells) as well as vascular and septal infiltrative changes with a predominance of lymphocytes and eosinophils. (Indicated by arrows) (**B**) Vaccinated mice depicting a discrete allergic inflammatory response characterized by a slight thickening of the bronchial wall. (**C**) Non-vaccinated non-challenged mice as the negative control.

To further assess the local inflammatory response after the allergen challenge, lung sections were subjected to histological examination, which showed a typical severe allergic response in the Th2 control group (Figure 6A). Thus, repeated allergen inhalation in sensitized mice led to significant chronic peribronchial inflammation characterized by eosinophil infiltration, subepithelial collagen deposition, and hyperplasia-metaplasia with mucin secretion. In contrast, the vaccinated mice showed a diminished allergic inflammatory response, with a striking difference as compared with the Th2 control group (Figures 6A,B). Overall, the vaccinated group behaved similarly to the negative control group, which was not subjected to the allergen challenge (Figure 6C).

### Effect of pre-existing immunity to *N. meningitidis*

This experiment was designed to assess the effect of pre-existing immunity to PL, both on the *N. meningitidis* and allergen-specific responses induced by the PL-containing allergen vaccine. Mice were primed with two doses of PL and later injected with three doses of the allergen vaccine (i.e., double immunization). Two control groups were used; one received only the allergen vaccine, and the other, only the PL priming (i.e., single-immunized groups).

**Figure 7:**
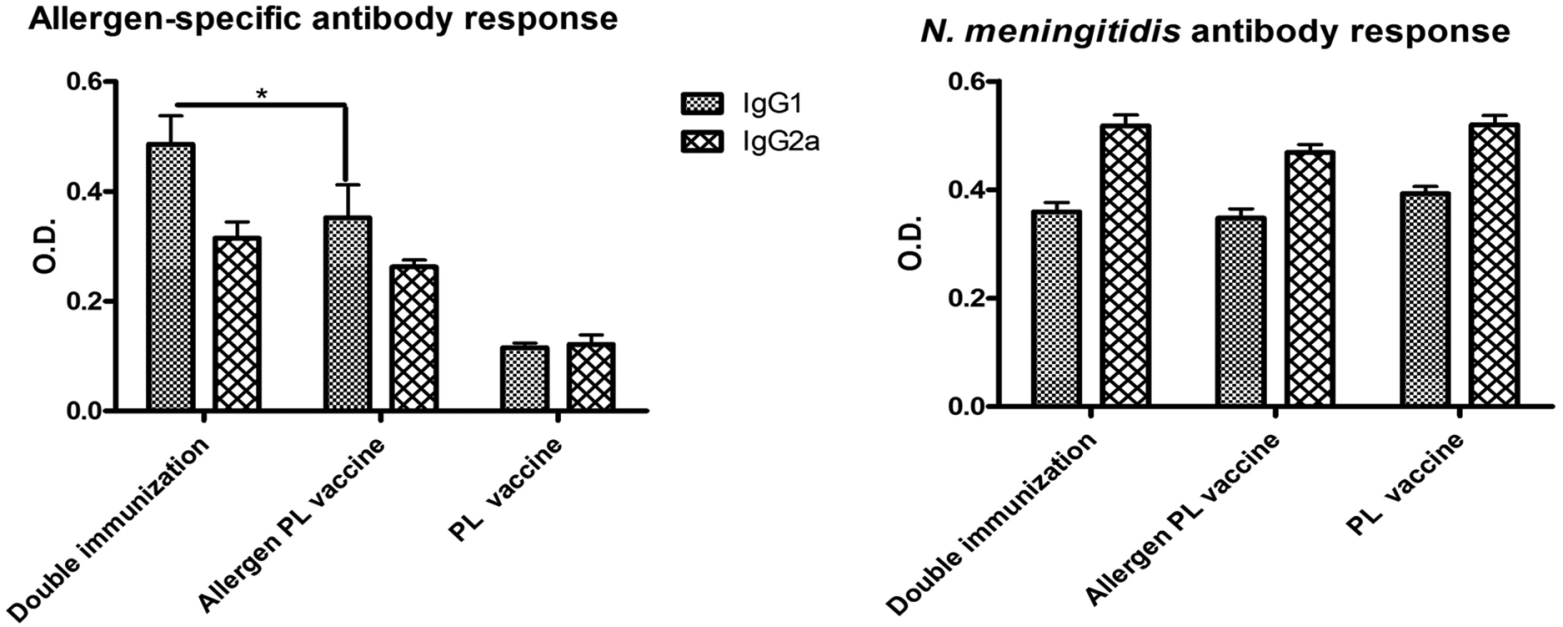
Influence of previous immunization with *N. meningitidis* PL into allergen-specific (left) and *N. meningitidis-*specific (right) IgG1 and IgG2a antibody response. Double vaccinated mice (i.e., mice administered first with PL and later with the allergen PL-adjuvanted vaccine) showed no change in anti-*N. meningitidis* antibodies as compared with single vaccinated mice (PL alone or allergen + PL alone), though a significant boost of IgG1 allergen-specific antibodies (*p < 0.05, ANOVA). Values are shown as mean ± SD from *n* = 10/group.

Both allergen-specific and *N. meningitidis* antibody responses showed a mixed IgG1/IgG2a pattern. However, interestingly, the allergen-specific IgG1 values in mice with double immunization were significantly greater (*p* < 0.05) as compared with mice that received only the allergen vaccine, suggesting the existence of a non-specific heterologous boosting effect by PL (Figure 7, left). In contrast, no homologous PL-boosting effect was noted since the *N. meningitidis*-specific antibody values (Figure 7, right) failed to present significant differences between double and single-immunized groups.

## Discussion

The suppression of Th2 immunity can occur as a consequence of induction of antigen-specific regulatory T cells; or as a consequence of immune deviation in favour of Th1 responses (26). Alum is known to promote the IgG4 blocking antibody responses (8, 9) and together with TLR agonists can be utilized to program the adaptive immune response enhancing the Th1 immunity (1). It has been postulated that targeting the innate immune system by combining multiple stimuli, such as those generated by TLR-dependent adjuvants improves the effectiveness of vaccine formulations (3).

In this work, a vaccine formulation containing allergens from *D. siboney* HDM and a combination adjuvant with PL of *N. meningitides* (a TLR2/4 ligand) and Alum was tested in a preventative experimental setting of respiratory allergy in mice. The administration of three doses of this vaccine rendered a clear anti-allergic protective effect, evidenced by the allergen exposure challenge. This response featured both dampening of systemic signs (IgE, eosinophils, IL-13 in lymph nodes), as well as, of local Th2 inflammatory response in the shock organ (IL4, IL5, eosinophil recruitment, mucus) together with a significant increase of the IgG1/IgE and IgG2a/IgE allergen-specific antibody ratios. The cytokine pattern was in agreement with the lung histology findings from animals subjected to the allergen challenge, which showed a remarkable decrease in the allergic inflammatory response in vaccinated animals as compared with sensitized control mice. These mice showed a severe inflammatory reaction characterized by eosinophil infiltration, goblet cells, neutrophils, and increased mucus secretion; as typical features of asthma pathology (27, 28).

The observed adjuvant effect here of the PL-containing formulation can be attributed to the TLR ligands contained in it, derived from the bacterial outer membrane, particularly to LPS. Other authors have tested TLR ligands in combination adjuvants; most of the studies combined a delivery system with the TLR agonists CpG ODN (a TLR9 ligand) or with MPL (a TLR4 ligand) (4). In a recent study, Mirotti et al. evaluated the effect of CpG + Alum (3); in a murine allergic model sensitized to OVA and found inhibition of OVA-induced lung allergic Th2 response. Another work, using a clinically relevant respiratory allergen (*Blomia tropicalis* extract) also showed the effect of CpG plus Alum, dampening the development of experimental asthma (29) in mice. In both works, the inhibition of pulmonary Th2 responses was associated with increased IL-10 production but not with IFN-γ production (in the lung). Moreover, an allergen vaccine containing CpG plus Amb a 1 (ragweed), even when failed in a Phase III clinical trial (30), showed clinical benefits for allergic rhinitis in a Phase II clinical trial (31) using only six pre-seasonal injections. In contrast to these findings, in our results, we detected both IFN-γ and IL-10 production in allergen-stimulated cultured lymphocytes extracted from the lymph nodes of challenged mice. This dual activation could be indicative of a mixed Th1/Treg response, or possibly, from a cellular population bearing both markers (32, 33).

It is well known that IL-10 plays an essential role as a regulatory molecule in both innate and adaptive immune responses (34). In a recent report (29), IL-10 was also considered critical for regulating the anti-allergic response to the HDM allergen *Blomia tropicalis* when applied with a combined CpG Alum adjuvant. One of the possible mechanisms explaining our and these results could be the induction of Tr1 peripheral lymphocytes with a regulatory phenotype, derived from Th1 cells capable to produce both IFN-γ and IL-10 (32, 33).

In agreement with our results, several works that have evaluated the LPS-derived MPL adjuvant, together with pollen allergens, even when using a different depot adjuvant (microcrystalline tyrosine) have shown clinical efficacy in clinical trials for pollen allergic rhinitis using only four pre-seasonal injections (5, 6, 35). The strategy to combine adjuvants with different immune signals has been poorly explored for AIT. One of the possible advantages of combining Alum with TLR ligands would be to induce or boost IgG-blocking antibodies, one of the well-documented mechanisms of AIT (36, 37). In line with our results, other authors have tested the CpG plus Alum combination showing a preventative anti-allergic effect in mice (29). Here, we have evidenced similar results but using a different HDM allergen and a TLR4 ligand instead of TLR9.

To assess not only the local effect but also the systemic outcome, in this work, we have measured IgE and IgG antibodies, as well as, the cellular response in lymphocyte cultures stimulated with the allergen. The vaccine has induced both IgG1 and IgG2a antibodies. The first one could be considered the mouse equivalent of the human IgG4, a class that is associated with Treg response and protection against allergic reaction (36, 37); while IgG2a antibodies are a feature of the Th1 response. The diminished IgG1/IgG2a ratio in vaccinated mice as compared with the Th2 control group supports a vaccine benefit over the Alum-adjuvanted formulation without PL suggesting that the vaccine was able to drive away the response from a typical Th2 allergic profile tilting it to Th1. In support of the adjuvant role of the TLR4 ligand, our results support that IgG2a antibodies response was dependent of the presence of PL in the combined formulation. The capacity of PL to induce IgG2a antibodies towards antigens included in that structure has been demonstrated previously by Pérez et al. (38). Nevertheless, in this study, this effect not only took place with bacterial antigens inserted into PL but also with antigens of very different nature (HDM allergens) adsorbed in a common solid phase: the aluminium gel. This fact also supports the use of Alum as a vaccine delivery system.

Upon allergen challenge, aimed to resemble the physiological exposure to the allergen, IL-13 and IgE, levels, as well as, blood eosinophils, have shown lower values as compared with the allergic control. These systemic effects, in addition to the local inflammatory changes, are in agreement with the induced IgG antibody response and are suggestive of a generalized anti-allergic protective response in real settings (39).

Mice models of respiratory allergy have strengths and limitations (40). A limitation of this work has been the use solely of the so-called preventative model, administering the vaccine in naïve, not sensitized mice. This model aimed to evaluate the primary effect of the adjuvant (PL) on the allergen-induced immune response. It is a common reductionist strategy that allows assessing the immune response exerted by the vaccine, without possible interference from the allergic status of the recipient. The use in this model of an allergen challenge by inhalation is intended to evaluate the functional interaction of the induced immune status with the allergen exposure by aerosols, reproducing the natural physiological route. Allergen particles can thus reach the lower respiratory tract triggering an allergic inflammatory reaction in this organ, which is relevant for resembling human allergic asthma. The findings using this “preventative” model could be predictive of efficacy for a preventative vaccination strategy in humans against HDM allergy, as a promising approach for managing highly prevalent allergic diseases (41). A previous work that explored the safety of this vaccine preparation has used also a “therapeutic” setting, i.e., administration of the product in previously sensitized mice mimicking the scenery of AIT in humans (19). However, further work is needed to develop models fully able to predict, not only safety but also AIT efficacy.

One of the goals of the present work was to evaluate the allergen-specific response induced by the vaccine in mice previously immunized to *N. meningitidis*. This setting resembles the real situation of humans naturally exposed to *N. meningitidis* or vaccinated against meningitis. Unexpectedly, the results showed that previous immunization to *N. meningitidis* tends to reinforce the IgG allergen-specific antibody response indicating a conditioning bystander effect, which could be attributed to the influence of the innate immunity on the adaptive response, since no sequence homology exists between the HDM and *Neisseria* proteins. The mechanism of this bystander effect could be based on the activation and maturation of DC by PL signals, recognized as pathogen-associated molecular patterns: LPS, porins, and peptidoglycans (15, 17). The PL is able also to induce in macrophages the production of soluble factors, such as TNFα, IL12, and nitric oxide (38). More research would be needed in order to clarify the precise mechanisms.

The identification of potential biomarkers is an important tool of translational medicine, particularly for AIT (39, 42, 43). Among proposed biomarkers, antibody class ratios are one of the most promising, since they can be easily evaluated in patients and can reflect the shift of Th2/Th1 balance during AIT (26, 44). In our study, both IgG1/IgE and IgG2a/IgE antibody ratios showed a significant contrast between vaccinated and allergic controls, and after the allergen challenge, this difference was maintained. These ratios show in one variable the balance between potentially blocking and effector components and it could be useful biomarkers to assess during clinical trials in humans, translated as IgG4/IgE and IgG1/IgE ratios.

In conclusion, the observed antibody and cellular responses reflect the anti-allergic preventative effect of this adjuvanted vaccine. Taken together, the findings suggest that this protective effect can be associated with the induction of a regulated Th1 response towards the allergen, characterized by the induction of IgG2a antibodies, IFN-γ, IL-10 in lymph nodes. The increase of IgG1/IgE and IgG2a/IgE antibody ratios could be useful biomarkers for translation into clinical trials in humans, which should be further evaluated in a therapeutic allergic model.

## Data Availability

The original contributions presented in the study are included in the article/Supplementary Material, further inquiries can be directed to the corresponding author/s.
